# 612. Coccidioidal Pulmonary Cavitation: A New Age

**DOI:** 10.1093/ofid/ofad500.678

**Published:** 2023-11-27

**Authors:** Lovedip Kooner, Austin Garcia, Akriti Kaur, Rupam Sharma, Virginia Bustamanate, Vishal Narang, Amir Berjis, Augustine Munoz, George R Thompson, Rasha Kuran, Royce H Johnson, Arash Heidari

**Affiliations:** Valley Fever Institute at Kern Medical, Bakersfield, California; Valley Fever Institute at Kern Medical, Bakersfield, California; Valley Fever Institute at Kern Medical, Bakersfield, California; Kern Medical Center, Bakersfield, California; Valley Fever Institute at Kern Medical, Bakersfield, California; Valley Fever Institute at Kern Medical, Bakersfield, California; Department of Surgery at Kern Medical, Bakersfield, California; Valley Fever Institute at Kern Medical, Bakersfield, California; University of California Davis Medical Center, Sacramento, CA; Kern Medical Center, Bakersfield, California; Kern Medical Center, Bakersfield, California; Dignity Health, Bakersfield, CA

## Abstract

**Background:**

The majority of literature on cavitary pulmonary coccidioidomycosis is from four decades ago which was prior to the advent of triazoles and focused on surgical treatment. This observational study is a comprehensive retrospective study of pulmonary cavitary coccidioidomycosis from patients at Valley Fever Institute at Kern Medical over the last 12 years. This observational study aims to explore the spectrum of coccidioidal cavities and the evaluation and management of those cavities.

**Figure:**
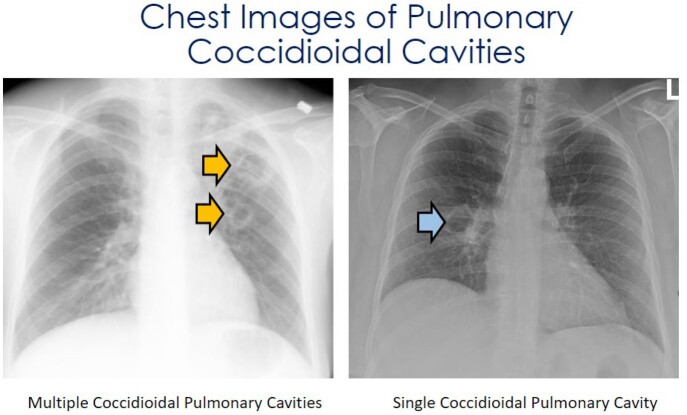

**Methods:**

IRB approved, retrospective review of electronic medical records of the Valley Fever Institute database was conducted. Demographics, comorbidities, types, and the number of cavities, complications, and medical and surgical treatment were gathered and compared to the literature. PubMed and Google Scholar were searched for cavitary pulmonary coccidioidomycosis

**Figure:**
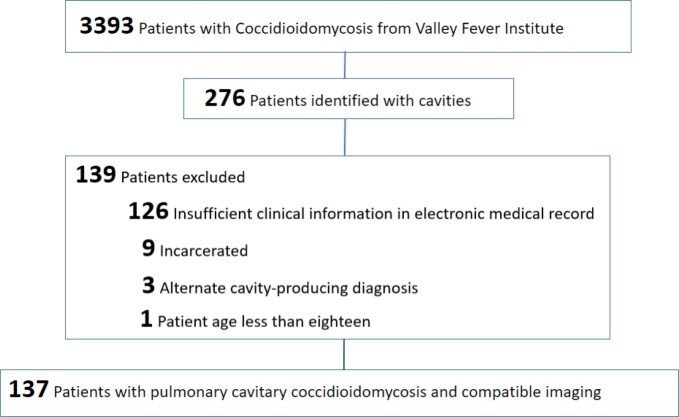

**Results:**

Of the initially 276 identified patients, 137 met the inclusion criteria. This study found 52 (37.2%) patients with hemoptysis. One case (0.7%) required radiologic intervention to occlude the bleeding vessel, and one (0.7%) case of hemorrhage required right upper lobe lobectomy. Nine (6.6%) cases exhibited a ruptured cavity. Eight of those cases had initial chest tube placement, of which three (3/8, 37.5%) did not require surgical intervention. Seven of 137 (5.1%) cases presented with a pleural effusion not associated with a cavity rupture. Five (3.7%) were due to primary coccidioidomycosis. Three of the coccidioidal effusions required therapeutic thoracentesis, and none required a chest tube or surgery. The mean duration of the initial antifungal treatment was found to be 563 days (n=80). In 35% (28/ 80) of them, a triazole was switched to another triazole for variable reasons, including treatment failure or side effects.

**Figure:**
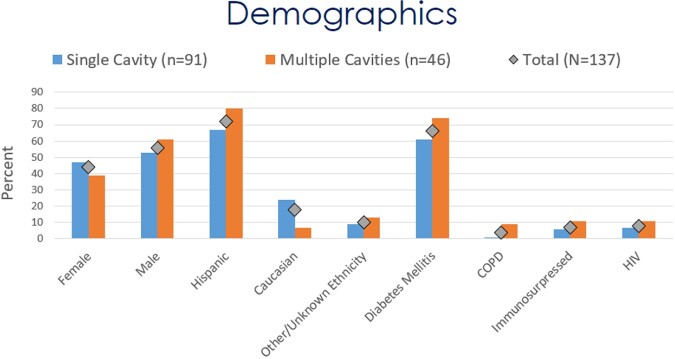

**Figure:**
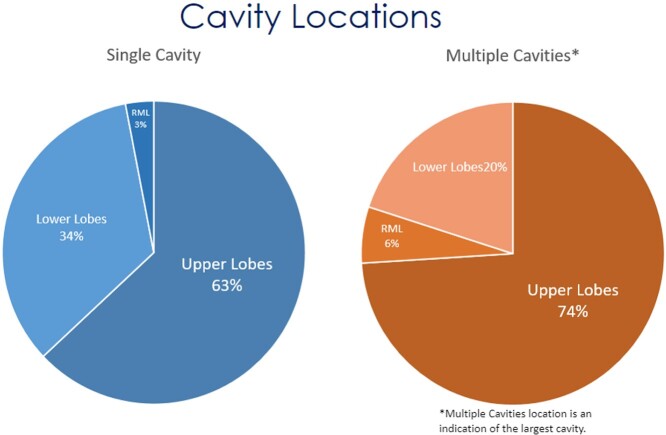

**Figure:**
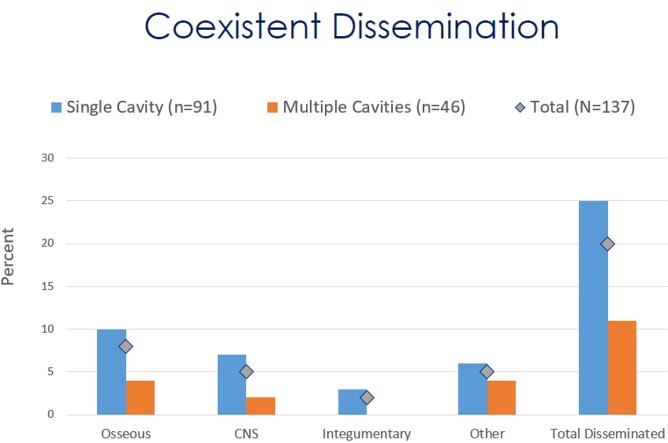

**Conclusion:**

Coccidioidal pulmonary cavitation remains a complex disease to evaluate and treat. This study's ethnic demographic differed from other cohorts. It also contradicts the notion that pulmonary coccidioidal cavitary disease and dissemination infrequently manifest in the same patient. In the present age of triazole therapy, indications and the need for surgery continue to decline. Further investigation needs to be conducted to evaluate medical therapy’s efficacy and long-term outcomes.

**Disclosures:**

**Rupam Sharma, PGY-1/MD**, Astellas: Grant/Research Support **George R. Thompson, III, MD**, Astellas: Advisor/Consultant|Astellas: Grant/Research Support|Cidara: Advisor/Consultant|Cidara: Grant/Research Support|F2G: Advisor/Consultant|F2G: Grant/Research Support|Mayne: Advisor/Consultant|Mayne: Grant/Research Support|Melinta: Advisor/Consultant|Melinta: Grant/Research Support|Mundipharma: Advisor/Consultant|Mundipharma: Grant/Research Support|Pfizer: Advisor/Consultant|Pfizer: Grant/Research Support

